# Hypodermic Medication

**Published:** 1869-02

**Authors:** S. F. Starley

**Affiliations:** Fairfield, Texas


Hypodermic Medication: By S. F. Starley, M. D., Fairfield, Texas.—The subject of Hypodermic Medication is one that has recently attracted much attention from that portion of the medical profession who endeavor to make their practice conform to the progressive stage of medical science; and few subjects now engaging the thoughts of investigators are likely to yield results more important to the practitioner of the healing art. Especially is this the case with regard to diseases of malarious origin, and of dangerous type—such as pernicious intermittent and congestive bilious fevers, where the stomach is too irritable to retain quinine, or from its diseased state will not readily absorb the remedy, so as to convey it into the circulation.

In such cases, the hypodermic syringe affords us a ready means of introducing the anti-periodic into the cellular tissue, from whence it readily filters through the thin walls of the capillaries and is swept at once into the blood currents, and commences its work of neutralizing and destroying the malarial poison—the deadly enemy, that has entered the circulation and taken possession of the citadel of life.

Given in this way, our remedy is subjected to none of the accidents that might interfere with its action if given by the mouth—such as loss of the dose by vomiting, injurious chemical actions that might readily take place in a disordered stomach, slowness of absorption, etc. But we know that the entire dose given in this way will take effect, and that a much smaller quantity is required to produce a given effect, than if administered in stomachic doses. From my own observations, I am inclined to think that the difference in dose should not be so great as is stated by some writers; but I am fully convinced that one grain of quinine, given hypodermically, is fully equal to thrice that quantity given by the mouth, and that it has the further advantage of much greater certainty of action—an advantage readily to be appreciated by every intelligent practitioner who has had much experience in the treatment of the malarious diseases of the South. In no disease with which I am acquainted, are the advantages of this mode of administering quinine more strikingly displayed than in the treatment of malarious hæmaturia; a congestive form of malarious fever, in which the morbid determination falls with such force upon the kidneys as to induce copious and even exhaustive hæmorrhage from these organs. The hæmorrhage recurring with each paroxysm of the fever, and in some cases continuing throughout the whole course of the disease.

In this disease, with which, unfortunately, physicians in this section of country are becoming but too familiar—the stomach is so irritable, and the bilious vomiting so constant that it is almost impossible in many of the cases to get the stomach to retain quinine long enough to affect the system; and yet, upon the prompt action of this remedy mainly depends the safety of the patient. In neuralgia, especially that form of the malady induced by long exposure to malarial influences, in which the chylopœtic viscera are always more or less diseased, the administration of morphia by the mouth constipates the bowels, and tends to increase the general derangement of the digestive apparatus. And yet, what conscientious physician would deny this "*Magnum Dei Donum*" to his patient, writhing under the malign inflictions of an excruciating neuralgia? Here the hypodermic syringe enables us to administer the soothing opiate to our patient without the disadvantage of inducing constipation or sickness. And here, too, it exerts in many cases a positively curative, as well as palliative action.

In sciatica—a most painful malady—there is, perhaps, no other treatment so effective as the hypodermic injection of atropine, from one-sixtieth to one fortieth of a grain, at intervals of three or four days. And I have no doubt but that in the course of future investigations other advantages of this method will be discovered and brought to the notice of the profession. At present it offers a profitable field of research for the earnest inquirer after medical truth, and enough is already known of its positive advantages to make it the duty of every practitioner to bring it to the aid of his patient in suitable cases. One advantage not to be overlooked, is, that it does not interfere with any other remedies that it may be deemed necessary to administer per oram. Hence there need be no waiting for the action of cathartics, as so many are in the habit of doing, before venturing to give quinine. This is no small item in treating malarious diseases that threaten to overwhelm the system before we can have time to put into practice the so-called preparatory measures of treatment. Here the hypodermic method enables us to secure the full effect of quinine at once, and thus procure its powerful aid in bringing the system into a condition favorable to the action of medicines addressed to the liver, bowels, kidneys, stomach, etc. The preparation of quinine I generally use is a solution containing thirty two grains of the remedy to the ounce of distilled water, with the addition of ten drops of sulphuric acid to dissolve the quinine. The syringe I use holds half a fluid drachm, so at each puncture I can give two grains of quinine, which I regard as being fully equal to six grains given by the mouth. When a larger dose is required I make two or more punctures, and thus avoid the local irritation that would result from introducing too much of the solution under the skin at one point. For some time I did not observe this precaution, and injected two or more syringes full through the same puncture. This would occasionally be followed by induration or abscess, and, in one case, where the patient was excessively anæmic, a small portion of the integument sloughed out around the point of puncture. But nothing of the kind has resulted in my practice since I have adopted the plan of only injecting a single syringeful of the solution at one point.

Of morphine, the best preparation is Magendie's solution, of which a quantity equal to one-eighth of a grain of morphine is a medium dose for an adult. And I will here say that I have never ventured to give morphine in this way to children. I think it would be dangerous, owing to their extreme susceptibility to the action of opiates.

I may conclude this paper by stating that I have used quinine hypodermically some hundreds of times, and with the exception of two or three cases of abscess, which gave little trouble, and the case of sloughing of a small portion of integument, above mentioned, have seen no ill result follow its use. But on the other hand its effects, when administered in this way, have been so prompt and so permanent, that several persons to whom I have given it now refuse to take the remedy by the mouth at all, and when quinine is prescribed, they insist upon having it injected under the skin.

It may seem to some that this subject is already too well understood to require much further notice in medical journals. But to such I would say, that this method of administering medicine is very far from being generally understood by physicians practicing in the country, and it is they who most frequently meet with the most alarming and most fatal attacks of malarious diseases, and need to be armed with every available means of coping with this deadly enemy of our race.

